# Effects of the peripherally acting μ-opioid receptor antagonist methylnaltrexone on acute pancreatitis severity: study protocol for a multicentre double-blind randomised placebo-controlled interventional trial, the PAMORA-AP trial

**DOI:** 10.1186/s13063-021-05885-3

**Published:** 2021-12-19

**Authors:** Cecilie Siggaard Knoph, Mathias Ellgaard Cook, Camilla Ann Fjelsted, Srdan Novovic, Michael Bau Mortensen, Liv Bjerre Juul Nielsen, Mark Berner Hansen, Jens Brøndum Frøkjær, Søren Schou Olesen, Asbjørn Mohr Drewes

**Affiliations:** 1 Mech-Sense, Department of Gastroenterology & Hepatology, Aalborg University Hospital, Aalborg, Denmark; 2 Department of Clinical Medicine, Aalborg University, Aalborg, Denmark; 3 Gastrounit, Copenhagen University Hospital Hvidovre, Hvidovre, Denmark; 4 Odense Pancreas Centre, HPB Section, Department of Surgery, Odense University Hospital, Odense, Denmark; 5 Digestive Disease Centre K, Bispebjerg Hospital, University of Copenhagen, Copenhagen, Denmark; 6 Mech-Sense, Department of Radiology, Aalborg University Hospital, Aalborg, Denmark

**Keywords:** Methylnaltrexone, Opioid antagonists, Drug antagonism, Acute pancreatitis, Treatment, Randomised controlled trial

## Abstract

**Background:**

Moderate to severe acute pancreatitis (AP) is associated with a high rate of complications and increased mortality, yet no targeted pharmacologic treatment currently exists. As pain is a dominant symptom in AP, patients are exposed to excess levels of both endo- and exogenous opioids, which may have harmful effects on the course of AP. This trial investigates the effects of the peripherally acting μ-opioid receptor antagonist (PAMORA) methylnaltrexone on disease severity and clinical outcomes in patients with moderate to severe AP.

**Methods:**

PAMORA-AP is a multicentre, investigator-initiated, double-blind, randomised, placebo-controlled, interventional trial, which will be conducted at four referral centres for acute pancreatitis in Denmark. Ninety patients with early-onset AP (pain onset within 48 h) as well as predicted moderate to severe disease (two or more systemic inflammatory response syndrome criteria upon admission) will be prospectively included. Subsequently, participants will be randomised (1:1) to intravenous treatment with either methylnaltrexone or matching placebo (Ringer’s lactate) during 5 days of admission. The primary endpoint will be the group difference in disease severity as defined and measured by the Pancreatitis Activity Scoring System (PASS) score 48 h after randomisation. Secondary endpoints include daily PASS scores; disease severity according to the Atlanta classification; quantification of need for analgesics, nutritional support, intravenous fluid resuscitation and antibiotics; duration of hospital admissions, readmission rates and mortality. Pain intensity and gut function will be self-reported using validated questionnaires. Exploratory endpoints include circulating levels of pro-and anti-inflammatory markers, polyethylene glycol recovery from the urine, circulating levels of blood markers of intestinal permeability, the prevalence of pancreatic complications on computed tomography (CT) scans, and colon transit time assessed using a CT-based radiopaque marker method.

**Discussion:**

This trial aims to evaluate the PAMORA methylnaltrexone as a novel targeted pharmacotherapy in patients with moderate to severe AP with the potential benefit of improved patient outcomes.

**Trial registration:**

ClinicalTrials.govNCT04743570. Registered on 28 January 2021. EudraCT 2020-002313-18.

## Administrative information

Note: the numbers in curly brackets in this protocol refer to SPIRIT checklist item numbers. The order of the items has been modified to group similar items (see http://www.equator-network.org/reporting-guidelines/spirit-2013-statement-defining-standard-protocol-items-for-clinical-trials/).

**Table:** 

Title {1}	Effects of the Peripherally Acting μ-opioid Receptor Antagonist Methylnaltrexone on Acute Pancreatitis Severity: Protocol for a Multicentre Double-blind, Randomised, Placebo-controlled Interventional Trial, the PAMORA-AP Trial
Trial registration {2a and 2b}.	ClinicalTrials.gov, Identifier: NCT04743570 EudraCT, Identifier: 2020-002313-18.
Protocol version {3}	Version 1.19, 2021.11.05
Funding {4}	Grant of 7.3 MIO DKK by the Novo Nordisk Foundation (#NNF190C0057331). Mech-Sense, Aalborg University Hospital, will cover the remaining expenses. The Novo Nordisk Foundation does not have any specific rights related to the publication of the results. No researchers involved in this trial have an economic interest in the Novo Nordisk Foundation or other financial supporters.
Author details {5a}	^1.^ Mech-Sense, Department of Gastroenterology & Hepatology, Aalborg University Hospital, Aalborg, Denmark. ^2.^ Department of Clinical Medicine, Aalborg University, Aalborg, Denmark ^3.^ Gastrounit, Copenhagen University Hospital Hvidovre, Hvidovre, Denmark ^4.^ Odense Pancreas Centre, HPB Section, Department of Surgery, Odense University Hospital, Odense, Denmark ^5.^ Digestive Disease Centre K, Bispebjerg Hospital, University of Copenhagen, Copenhagen, Denmark ^6.^ Mech-Sense, Department of Radiology, Aalborg University Hospital, Aalborg, Denmark
Name and contact information for the trial sponsor {5b}	Asbjørn Mohr Drewes, Mech-Sense, Department of Gastroenterology & Hepatology, Aalborg University Hospital, 9000 Aalborg, Denmark. Mail: amd@rn.dk, Phone: +45 97663562
Role of sponsor {5c}	The trial was conceived and initiated by Professor Asbjørn Mohr Drewes and professor Søren Schou Olesen. As sponsor, AMD has the ultimate authority over trial design, collection, management, analysis, interpretation of data, writing of the report, and the decision to submit for publication.

## Introduction

### Background and rationale {6a}

Acute pancreatitis (AP) is a frequent gastrointestinal disease [1, 2]. Approximately 20% of patients with AP develop moderate to severe disease, associated with a high rate of complications and excess mortality [3, 4]. The mechanisms behind the development of AP are not fully understood. Still, the inflammation presumably starts in the acinar cells with premature activation of trypsinogen to trypsin, ultimately leading to autodigestion of the tissue [5]. However, several other mechanisms may be involved in further progression of the intrapancreatic pro-inflammatory processes. Activated pancreatic enzymes and the associated apoptosis stimulate the production of pro-inflammatory mediators, which promotes leucocytes to migrate into the interstitial spaces. The infiltration of immune cells further increases the release of cytokines and chemokines, causing an inflammatory cascade. Hence, the localised inflammation within the pancreas can progress to a systemic inflammatory response syndrome (SIRS) associated with multiorgan failure and increased mortality [6–8]. No pharmacological treatment has proven effective in preventing the progression of intrapancreatic inflammation and subsequent SIRS. Thus, the management of AP is currently supportive with fluids, analgesics, and nutrition, thereby supporting organ functions. Secondary treatment targets complications such as pancreatic necrosis, organ failure and infections [9].

**Fig. 1:**
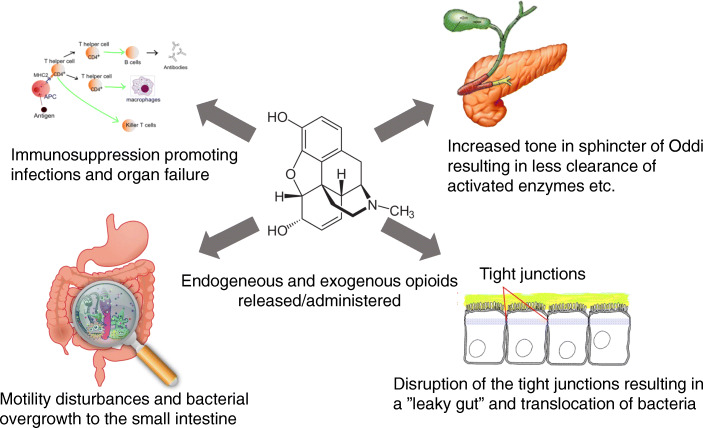
Effects of opioids on the gastrointestinal tract and immune system

Pain and systemic inflammation are cardinal features of AP, both leading to endogenous opioid release [10]. Furthermore, exogenous opioid medicines are often part of the mainstay for pain management [11]. Opioid administration is known to cause opioid-induced bowel dysfunction primarily by binding μ-opioid receptors in the enteric nervous system [12, 13]. Thus, opioids promote dysmotility and prolonged gut transit time, which together can cause small intestinal bacterial overgrowth [13]. Furthermore, opioids may increase intestinal permeability, resulting in the translocation of bacteria from the gut [14] to the peripancreatic tissue and systemic circulation. Potentially, translocation of bacteria may lead to local and systemic infections, which further may be facilitated by opioid-induced immunosuppression [15, 16]. Opioids also affect the pancreas directly by decreasing fluid secretion in the pancreatic duct system and increasing the frequency of contractions in the sphincter of Oddi [13, 17]. This may lead to decreased wash-out of intrapancreatic activated enzymes and thus worsen autodigestion of the tissue and subsequent inflammation of the pancreas. These potentially deteriorating effects of opioids on the disease course in patients with AP are summarised in Fig. 1.

Previous research has shown that treatment with peripherally acting μ-opioid receptor antagonists (PAMORAs) counteracts opioid-induced adverse effects on the gastrointestinal tract by normalising gut motility patterns, decreasing gut transit time, relaxing sphincters, including the sphincter of Oddi, increasing the intestinal intraluminal water content and enhancing flow in the pancreatic duct system [13, 18–23]. Furthermore, treatment with PAMORAs may potentiate the immune response and reduce inflammation [22, 24]. PAMORAs do not cross the blood-brain barrier and have a much higher affinity towards peripheral μ-opioid receptors than opioids themselves. Potentially, treatment with PAMORAs will therefore counteract the putative harmful effects of opioids in patients with AP without compromising analgesia. Still, treatment with PAMORAs has not previously been examined in a clinical trial concerning patients with AP. We hypothesise that, compared to placebo, acute treatment with the PAMORA methylnaltrexone will reduce disease severity and improve clinical outcomes in patients admitted with moderate to severe AP.

### Objectives {7}

The primary objective of this trial is to investigate the potential beneficial effects of treatment with the PAMORA methylnaltrexone on disease severity in patients admitted with AP. Secondarily, we will evaluate the effects of treatment with methylnaltrexone on clinical outcomes, patient symptoms, health resource utilisation, systemic inflammation, gut motility, intestinal permeability and pancreatic complications. Accordingly, this trial will generate important new clinical and pathogenic information for the use of methylnaltrexone in the management of AP.

### Trial design {8}

PAMORA-AP is a multicentre, investigator-initiated, double-blind, 1:1 randomised, placebo-controlled interventional, parallel-group, superiority trial.

## Methods: Participants, interventions, and outcomes

### Study setting {9}

PAMORA-AP will be conducted at four referral centres for acute pancreatitis in Denmark (Aalborg University Hospital, Odense University Hospital, Copenhagen University Hospital Hvidovre, and Bispebjerg Hospital). Patients with AP will primarily be included in the emergency department upon admission as inclusion and randomisation must be done within 48 h of symptom onset.

### Eligibility criteria {10}

#### Inclusion criteria

- Age between 18 and 80 years
- Ability to understand spoken and written Danish
- Written informed consent
- Expected to comply with and complete the trial protocol
- Verified diagnosis of AP according to the criteria given within the revised Atlanta classification [25]
- Predicted moderate to severe AP based on two or more SIRS criteria upon admission [6]
- For fertile female participants: negative pregnancy test prior to randomisation and contraception during the trial period

#### Exclusion criteria

- Definitive chronic pancreatitis according to the M-ANNHEIM criteria [26]
- Known allergy towards methylnaltrexone
- Known or suspected major stenosis or perforation of the intestines
- Known or suspected gastrointestinal cancers
- Pre-existing renal insufficiency (defined as habitual estimated glomerular filtration rate < 45 ml/min)
- Severe pre-existing comorbidities (assessed by investigator upon inclusion)
- Severe non-pancreaticobiliary infections or sepsis caused by non-pancreaticobiliary disease
- Child-Pugh class B or C liver cirrhosis
- Females that are currently lactating

### Who will take informed consent? {26a}

Potential participants will be informed about the trial by medical doctors or trained research staff, with good clinical practice (GCP) authorisation and knowledge about randomised-controlled trials and AP. Before giving informed consent, sufficient time to consider participation will be provided. Only medical doctors will evaluate in- and exclusion criteria prior to inclusion.

### Additional consent provisions for collection and use of participant data and biological specimens {26b}

In signing the informed consent, participants will be separately asked whether they want to contribute to the establishment of a biobank for blood and urine samples.

## Interventions

### Explanation for the choice of comparators {6b}

Currently, the treatment of AP is merely supportive or directed against complications such as infections, necrosis or organ failure [9, 27]. Thus, we chose to conduct a randomised placebo-controlled clinical trial to demonstrate the superiority of PAMORAs over placebo. Ringer’s lactate was chosen as the placebo as it is included in the standard of care for patients with AP according to the International Association of Pancreatology/American Pancreatic Association (IAP/APA) guidelines.

### Intervention description {11a}

Following randomisation, 0.15 mg/kg methylnaltrexone [28–30] or a corresponding volume of matching placebo (Ringer’s lactate) will be dissolved in 1000 ml Ringer’s lactate solution. This investigational medicinal product (IMP) solution will subsequently be administered daily as a continuous intravenous infusion over 24 h on an infusion pump and repeated for 5 days after randomisation. In accordance with the summary of product characteristics for methylnaltrexone [30], the continuous infusion of IMP will be started no later than 24 h after preparation. As methylnaltrexone is sensitive to light, the solution will be kept protected from light from the time of preparation until the time of infusion start.

### Criteria for discontinuing or modifying allocated interventions {11b}

A trial participant should terminate intervention if they wish to do so or if the investigator judges it necessary due to medical reasons. Furthermore, trial participants ready for discharge before day 5 will discontinue intervention upon discharge. The daily dose of IMP is fixed according to weight on admission.

### Strategies to improve adherence to interventions {11c}

Interventions will be administered at fixed time points and subsequently registered within the electronic case report form (eCRF). The IMP and solution hereof will be administered by medical personnel only, and a detailed account of the volume administered will be kept for each trial participant within the eCRF.

### Relevant concomitant care permitted or prohibited during the trial {11d}

Trial participants will receive standard of care, which follow the IAP/APA guidelines for managing AP [9] as prescribed by their treatment-responsible physician. This treatment includes supportive therapy with intravenous fluid therapy (Ringer’s lactate 5–10 ml/kg per hour), enteral feeding, and analgesics. Standard of care for patients with AP may also include biliary tract management (e.g. endoscopic retrograde cholangiopancreatography, magnetic resonance cholangiopancreatography, endoscopic ultrasound, endoscopic sphincterotomy), invasive treatment of necrotising pancreatitis (e.g. percutaneous catheter drainage, endoscopic transluminal drainage, necrosectomy) or prophylactic cholecystectomy. Concomitant medication and care will be registered within the eCRF. No patient care is prohibited for trial participants.

### Provisions for post-trial care {30}

All adverse events or reactions will be followed until stabilised or resolved. Clinical responsibility lies with the hospitals involved in the trial. Furthermore, patient insurance of the relevant trial site will cover trial participants. This insurance includes coverage of any intervention-related harms.

### Outcomes {12}

#### Primary outcome

- The difference in pancreatitis activity scoring system (PASS) score (described in detail in {18a}) between the methylnaltrexone group and the placebo group 48 h after randomisation

#### Secondary outcomes

- The difference in daily PASS scores between the treatment groups during treatment and at 14-day follow-up (methylnaltrexone vs placebo)
- Differences between the groups in disease severity according to the revised Atlanta classification [25]
- Differences between groups in pain intensity and gut function evaluated daily by questionnaires during treatment and at 14-day follow-up: the modified Brief Pain Inventory - short form [31], Bristol Stool Form Scale [32], and (iii) Gastrointestinal Symptom Rating Scale [33]
- Differences between the groups in the following clinical outcome parameters assessed daily during treatment and at 14-day follow-up: quantification of analgesics (separated into opioids and non-opioids), need for nutritional support, need for intravenous fluid resuscitation or antibiotics
- The difference in utilisation of health resources (invasive treatments, intensive care, and readmission rates) between the groups
- Differences between groups on the duration of hospital admissions and mortality. These will be determined retrospectively 30 and 90 days after admission using the patients’ medical records

#### Explorative outcomes

- The difference between groups in daily levels of circulating pro- and anti-inflammatory markers during treatment and at 14-day follow-up (including C-reactive protein, interleukin-6, interleukin-8, interleukin-18, tumour necrosis factor-α, and cluster of differentiation 163)
- The difference in daily levels of circulating blood markers of intestinal permeability [34] between the groups during treatment and at 14-day follow-up
- The difference in intestinal permeability between the groups measured from 48 to 72 h after randomisation using the oral polyethylene glycol (PEG) 400/4000 test (described in detail in {33}) [35]
- The difference in gut transit time assessed by a CT-based radiopaque marker method on day 5 (+/− 1 day) after randomisation (described in detail in {18a}) between the groups [36]
- The group difference in the prevalence of pancreatic complications (e.g., oedema, fluid collections, and necrosis) assessed and quantified by contrast-enhanced CT on day 5 (+/− 1 day) after randomisation [25]

### Participant timeline {13}

The participant timeline is shown in Table 1.

**Table 1:**
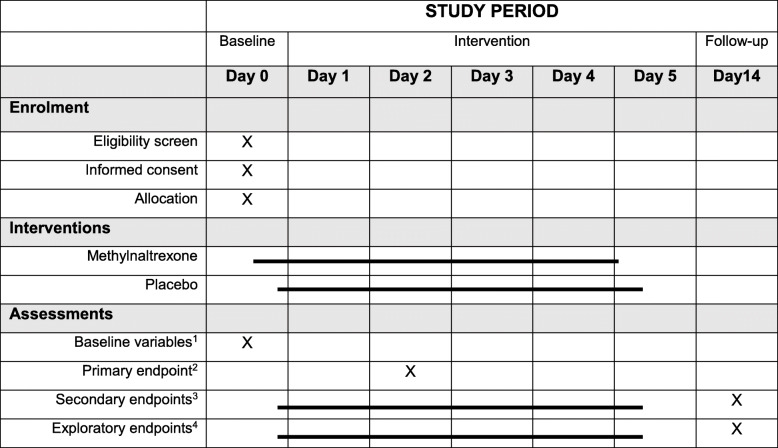
Participant timeline

^1^Baseline variables: sex, age, height, weight, time of symptom onset, time of hospitalisation, weekly alcohol consumption, smoking status, Charlson comorbidity index-score [37]

^2^Primary endpoint: Pancreatitis activity scoring system (PASS) score after 48 h

^3^Secondary endpoints: daily PASS scores; disease severity according to the Atlanta classification; daily questionnaires: The modified Brief Pain Inventory short form [31], Bristol Stool Form Scale [32], and Gastrointestinal Symptom Rating Scale [33]; clinical outcome parameters: quantification of need for analgesics, nutritional support, intravenous fluid resuscitation and antibiotics; health resource utilisation: invasive treatments, intensive care, readmission rates and duration of hospital admissions; and mortality

^4^Exploratory outcomes: daily levels of circulating pro- and anti-inflammatory markers; daily levels of circulating blood markers of intestinal permeability; polyethylene glycol 400/4000 test [35]; gut transit assessed using a CT-based radiopaque marker method [36] and pancreatic complications: assessed and quantified by contrast-enhanced CT according to the revised Atlanta criteria [25]

### Sample size {14}

We calculated that 41 trial participants would be needed per group to detect a difference in the PASS score (described in detail in {18a}) of 25 points after 48 h with a within-group standard deviation of 40 points (38), 80% power, and a 2-sided alfa level of 0.05. As we anticipate loss to follow-up on the primary outcome due to unforeseen events during admission or early admission, the sample size is set at 45 participants per group, and we plan to include a total of 90 patients in the trial. Statistical methods to handle loss to follow-up on both primary and secondary outcomes are described in {20c}.

### Recruitment {15}

Patients admitted with AP will be identified and contacted by trial personnel upon admission. Trial personnel will identify eligible patients by daily contact with the staff at the emergency departments. AP is a relatively common disease. We expect a total of 10 patients with AP to be admitted and subsequently screened at our four inclusions sites each week.

## Assignment of interventions: allocation

### Sequence generation {16a}

The Hospital Pharmacy at Herlev Hospital, Denmark, will conduct randomisation in random block sizes without stratification (block-randomisation) using statistical software approved for this purpose, e.g. from the website www.randomization.com. Dropouts (treatment with IMP less than 48 h) will be replaced by new subjects, and a mirror-randomisation will be performed.

### Concealment mechanism {16b}

Labelling will be performed by the Hospital Pharmacy at Herlev Hospital, according to Annex 13 of the Good Manufacturing Practice guidelines of the International Conference on Harmonisation-GCP guidelines and local law [38]. The IMP will be delivered directly to the respective trial sites by the Hospital Pharmacy at Herlev Hospital in vials labelled with the randomisation number corresponding to the allocation and the information that it is intended for use in a clinical trial only. Each vial contains 0.6 ml of transparent fluid corresponding to 12 mg methylnaltrexone or matching volume of Ringer’s lactate.

### Implementation {16c}

A list of randomisation numbers is devised by the Hospital Pharmacy at Herlev Hospital and provided to trial personnel. After inclusion, a randomisation number is assigned to the individual trial participant as instructed by the Hospital Pharmacy.

## Assignment of interventions: Blinding

### Who will be blinded {17a}

The trial is double-blinded, and Herlev Hospital Pharmacy will perform the blinding. This way of blinding will ensure that trial personnel and participants are prevented from knowing information regarding the allocation. Care providers will also be blinded as the IMP will have the same appearance regardless of assignment.

### Procedure for unblinding if needed {17b}

A medical emergency (e.g. suspected unexpected serious adverse reaction) may necessitate information on the treatment allocation. Thus, sealed envelopes containing the assignment of each trial participant will be available at all trial centres day and night throughout the trial period. These envelopes will be securely stored, only accessible to delegated trial personnel. Furthermore, the other trial centres can always contact the primary trial centre in case unblinding is needed. After unblinding, the reason for breaking the code, date, and signature must be stated on the envelope.

## Data collection and management

### Plans for assessment and collection of outcomes {18a}

As part of the trial, participants will have the PASS score rated daily to document and monitor disease activity. PASS is a validated assessment tool for AP activity based on 5 clinical parameters: organ failure, SIRS, abdominal pain, morphine equivalent doses, and tolerance to solid diets [39]. It was developed to quantify disease activity in patients with AP and has proven useful when monitoring disease severity and predicting clinical outcomes in patients admitted with AP [40, 41]. Organ failure will be assessed according to the Modified Marshall scoring system as defined in the Revised Atlanta criteria [25]. The ratio between the partial pressure of oxygen in arterial blood and the fraction of inspired oxygen is used to assess the respiratory system. Not all participants included in this trial are expected to have partial pressure of oxygen in arterial blood values available. Thus, we will use the ratio between peripheral capillary oxygen saturation and the fraction of inspired oxygen instead, as previous studies have shown that these two ratios correlate well [42, 43].

Clinical outcomes and utilisation of health resources are documented using validated questionnaires and medical records. Participants will be asked to fill out three questionnaires daily. The modified Brief Pain Inventory - short form [31] subjectively assesses pain intensity and impact on daily functions. Bristol Stool Form Scale [32] assesses stool consistency and frequency. Gastrointestinal Symptom Rating Scale [33] subjectively quantifies patient symptoms within 5 symptom groups: reflux, abdominal pain, indigestion, diarrhoea and constipation. From the trial participants’ medical records, the following will be registered: (i) presence of local and systemic complications as well as organ failure (transient or persistent), used to classify the severity of AP according to the revised Atlanta criteria [25]; (ii) need for intensive care unit admission; (iii) need for analgesics; (iv) nutritional support; (v) need for intravenous fluid resuscitation or antibiotics; (vi) invasive treatments; (vii) duration of hospitalisation; (viii) readmissions; and (ix) mortality.

An abdominal contrast-enhanced CT trial will be performed at inclusion and after the conclusion of treatment at day 5 (+/− 1 day) to document the presence and extent of pancreatic inflammation, fluid collections, and necrosis according to the definitions given in the revised Atlanta criteria [25]. As an integrated part of the CT exam at day 5 (+/− 1 day), trial participants’ gut transit time will be assessed using radiopaque markers administered orally on day three after randomisation [36]. All participants will be invited for a follow-up visit on day 14 (+/− 2 days), where the outcomes assessed during hospital admission will be reassessed.

### Plans to promote participant retention and complete follow-up {18b}

IMP administration, blood sample results, vital signs, and concomitant medication will be registered within the trial participants’ medical records as part of their standard treatment, and subsequently transferred to the eCRF. Data handling in relation to early discontinuation of IMP will be stratified according to the following:

- < 48 h of treatment: All data collection will be terminated upon discontinuation of IMP and participant is regarded as dropout.
- ≥ 48 h of treatment, but discharge before day 5: Participant will be lost to follow-up on the following outcomes: daily PASS scores, blood samples, vital signs, quantification of need for analgesics, nutritional support, intravenous fluid resuscitation and antibiotics. The participant will be asked to complete questionnaires at home and they will be offered the follow-up CT scan in an outpatient setting on day 5 (+/− 1 day). Furthermore, they will be invited to participate in the 14-day follow-up and readmission rates as well as mortality will be registered retrospectively.

### Data management {19}

Delegated trial personnel at each trial centre will register data in the eCRF using the electronic data capture tool REDCap (Research Electronic Data Capture, version 10.6.26) hosted by the organisation of The North Denmark Region. REDCap is a secure browser-based software, which meets all regulatory safety requirements [44, 45]. Data recording will begin when a participant is included and will occur gradually to the end of the trial. A detailed record of any corrections will be kept within REDCap.

### Confidentiality {27}

The collection of sensitive personal data will be conducted by delegated site staff and kept securely at Aalborg University Hospital, Department of Gastroenterology and Hepatology, for a minimum of 5 years after the trial has ended. Storage will be electronically in REDCap and physically at the relevant inclusion site within a locked cabinet placed in a locked room. After 5 years, all electronically or physically stored data will be anonymised or destroyed.

### Plans for collection, laboratory evaluation, and storage of biological specimens for genetic or molecular analysis in this trial/future use {33}

Blood samples will be drawn at baseline, daily at fixed time points for 5 consecutive days during treatment with the IMP and at day 14 follow-up. A part of these samples will be analysed immediately as standard of care for patients with AP (e.g. C-reactive protein, white blood cell count, serum creatinine, amylase). The remaining samples will be kept in a biobank to measure levels of circulating pro- and anti-inflammatory markers and circulating blood markers of intestinal permeability. Intestinal permeability will furthermore be evaluated using the oral PEG 400/4000 test [35]. Following ingestion of a PEG solution containing 5 g PEG 400 and 5 g PEG 4000 dissolved in 100 ml water, trial participants will have their urine collected for 24 h. The small size molecules (PEG 400) traverse the intestinal barrier freely, independent of barrier function loss, whereas the large size molecules (PEG 4000) only cross the intestinal wall and becomes detectable in urine in case of intestinal barrier function loss. Upon collection, blood and urine samples will be homogenised and stored at − 80 °C at each inclusion site. Blood and urine samples will be batch-analysed after the conclusion of the trial at a central laboratory. After analysis, remaining urine and blood samples will be stored in a biobank for future research purposes. All samples will be destroyed 15 years after the conclusion of the trial at the latest.

## Statistical methods

### Statistical methods for primary and secondary outcomes {20a}

For the primary analysis of PASS, a repeated measures linear mixed-effects model will be used, and terms for the treatment group, assessment time point, and the interaction of treatment with assessment time point will be included. The difference in PASS scores between the groups 48 h after randomisation is considered the primary efficacy parameter. Furthermore, summary statistics and trend curves of PASS scores will be provided for the individual time points. The primary analysis will be by intention-to-treat. Trends in secondary endpoints repeatedly assessed during the treatment period (e.g. PASS scores, clinical outcome parameters and levels of circulating cytokines) are analysed using a linear mixed-effects model as for the primary endpoint. Single time point outcomes (baseline characteristics, CT features, and measurements of gut permeability and gut transit time) will be compared using Student’s *t* test or non-parametric analysis for continuous data as appropriate. Binary outcomes will be analysed using a *χ*^2^ test or Fisher’s exact test as appropriate. All secondary endpoints will be analysed per protocol.

### Interim analyses {21b}

No interim analysis has been planned.

### Methods for additional analyses (e.g. subgroup analyses) {20b}

Subgroup and covariate analyses will be performed if differences in patient or treatment subgroups are evident and deemed clinically relevant.

### Methods in analysis to handle protocol non-adherence and any statistical methods to handle missing data {20c}

The Last-Observation-Carried-Forward method will be employed in case of early hospital discharge or other reasons for missing values.

### Plans to give access to the full protocol, participant level-data and statistical code {31c}

The anonymised data will be available to other researchers through relevant public databases such as Zenodo or FigShare [46] after the trial has ended.

## Oversight and monitoring

### Composition of the coordinating centre and trial steering committee {5d}

Mech-Sense, Aalborg University Hospital, will be the coordinating centre—thus taking overall responsibility for the conduct of the trial. Mech-Sense is an interdisciplinary research group with long records in clinical and experimental research in pain, pancreatitis, opioids, opioid antagonists, and imaging. Furthermore, Mech-Sense has an extensive network and significant experience with leadership of multicentre studies. We will be available for day-to-day support for all sites for the duration of the trial. The trial steering committee consists of one or two members from each site and has been involved in planning the trial as well as evaluating progress in carrying out the trial. In this regard, all sites involved in the trial will participate in virtual monthly meetings to update on trial progress at each site. Following these meetings, a summary will be devised and sent to each site as a monthly newsletter. A detailed plan for sponsor oversight with regular supervision of each site and collaborators has been devised. Furthermore, it will be documented that this plan is followed.

### Composition of the data monitoring committee, its role and reporting structure {21a}

The trial will be monitored by the GCP units in Copenhagen, Aalborg, Aarhus and Odense. Thus, all sites will be appointed an independent monitor who will visit regularly to ensure compliance and completion of the protocol as well as concordance with GCP standards and Danish regulations. A thorough inspection of source documents will ensure that the data collected are consistent and accurate. Investigators will provide direct access to source documents during monitoring, auditing, and inspection by the GCP units as well as the Danish authorities if required. Furthermore, a gastroenterologist/abdominal surgeon from outside the involved institutes will conduct external safety monitoring annually.

### Adverse event reporting and harms {22}

Expected and unexpected adverse events (AEs) and adverse reactions (ARs) will be registered from the first administration of IMP until 45 h after discontinuation of the IMP. Forty-five hours corresponds to 5 times the half-life of methylnaltrexone [30]. AEs and ARs will be documented within the eCRF and included in the final report registered with EudraCT unless they fulfil the criteria described below. Specific symptoms and laboratory result deviations frequently associated with AP are expected in trial participants. Thus, the following will not be reported as AEs or ARs: abdominal pain, nausea, vomiting, and certain laboratory result deviations (e.g. elevated plasma amylase/lipase, elevated C-reactive protein, elevated serum liver enzymes or bile acids, elevated serum creatinine, elevated blood sugar levels and elevated white blood cell count) [47]. AEs and ARs fulfilling the criteria for serious and ARs fulfilling the criteria for serious and unexpected according to the definitions given by the Danish authorities will be reported to sponsor by the investigator within 24 h. Exempt from this are the most common complications associated with AP – septic shock, kidney failure, and acute respiratory distress syndrome—which will be reported within seven days. All suspected unexpected serious adverse reactions will be reported ongoingly within the deadlines given by the Danish authorities, whereas other serious AEs and ARs will be reported annually.

### Frequency and plans for auditing trial conduct {23}

As described in {21a}, an independent GCP monitor will contact and subsequently visit each trial site regularly. Source documents, eCRFs, and trial participants’ medical records will be made available for inspection, and monitor will ensure that participants’ personal information is securely stored as described in {27}. Furthermore, the GCP monitor will ensure that participants have given informed consent prior to any trial-specific procedures and that data is collected accurately according to the protocol. Afterwards, a report of potential problems discovered at monitoring will be devised. It is the responsibility of the sponsor to go through this report and solve potential problems.

### Plans for communicating important protocol amendments to relevant parties (e.g. trial participants, ethical committees) {25}

Updates on protocol modifications will be disseminated from sponsor to relevant personnel ongoingly via a shared electronic database and at monthly virtual meetings between all participating in the trial as described in {5d}. Furthermore, all substantial protocol modifications will be submitted to the Danish authorities according to regulations. Any deviations from the protocol will be fully documented using a breach report form and subsequently assessed for severity by the sponsor (AMD). Protocol modifications will also be updated in the clinical trial registry ClinicalTrials.gov.

### Dissemination plans {31a}

Results, positive as negative or inconclusive, will be released to the public, published in peer-reviewed scientific journals and presented at relevant international scientific conferences. Furthermore, the trial results will be posted via EudraCT and ClinicalTrials.gov. Results will also be shared with the Danish authorities according to regulations.

## Discussion

Moderate to severe AP carries a high rate of complications and mortality as high as 40–50% in patients with persistent organ failure and infected pancreatic necrosis [3, 4]. Furthermore, 10% of patients progress towards chronic pancreatitis after just one occurrence of clinical AP, and this rate may increase to 36% in the presence of recurrent AP [48]. Thus, AP may lead to disablement for the individual patient and significant costs for society. As no specific pharmaceutical treatment for AP exists, research within new treatment modalities is an unmet need.

In this multicentre, double-blinded, randomised, placebo-controlled interventional trial, we investigate whether the PAMORA methylnaltrexone can improve disease severity and clinical outcomes in patients admitted with predicted moderate to severe AP. Previous data suggest that opioids exacerbate the course of AP by affecting both the gastrointestinal tract (primarily through μ-opioid receptors) and the immune system [12–17]. In line with this, eluxadoline, a mixed μ-opioid receptor agonist and δ-opioid receptor antagonist, which is used to treat irritable bowel syndrome, may trigger AP, particularly for patients with a previous history of cholecystectomy [49]. As shown by our group and others, PAMORAs have the potential to counteract such opioid-induced gastrointestinal changes [18–24]. Interestingly, preclinical models have shown that morphine increases AP-associated pancreatic necrosis, and this is antagonised in μ-opioid receptor knockout mice or in the presence of the PAMORA naltrexone [50].

Disease severity will be monitored using the PASS score, which quantifies disease activity in patients with AP based on a 12-h observation period and is validated for this purpose [39–41]. The PASS score has the benefit of being simple to calculate and based on parameters accessible through standard patient care. Some elements of the PASS score are at risk of high inter-individual variability (e.g. abdominal pain, morphine equivalent dose). Thus, we will also document several clinical outcomes (e.g. need for intravenous fluids and analgesics), estimate health resource utilisation (e.g. duration of admission and mortality) and classify the severity of AP according to the revised Atlanta criteria [25] for each patient. To identify patients at risk of moderate to severe AP, we will only include patients who fulfil two SIRS criteria or more upon admission. SIRS has previously shown an association with severity and mortality in patients with AP [6–8]. This criterion may raise the issue of reduced inclusion rates, which we plan to alleviate by recruiting at several sites. By recruiting at several sites, we also decrease the risk of selection bias, whereas Danish centres only will ensure homogenous patient characteristics and management.

To provide novel mechanistic insights into the role of opioids and the potential benefits of opioid antagonism in AP, we will evaluate key features of the gastrointestinal function and the immune response, which are known to be affected by opioid administration, as summarised in Fig. 1. Thus, we will evaluate the motility and integrity of the intestines through measures of gut transit time and intestinal permeability. In this regard, we expect treatment with PAMORAs to decrease gut transit time and alleviate intestinal leakage, thereby reducing the risk of intestinal bacteria spill-over into the systemic circulation. The level of systemic inflammation will be evaluated by measuring levels of circulating pro-and anti-inflammatory cytokines. Finally, the inflammation within the pancreas will be evaluated based on diagnostic imaging according to the definitions given in the revised Atlanta criteria [25]. We expect PAMORAs to relax the sphincter of Oddi and enhance flow within the pancreatic duct system, potentially abating the progression of intrapancreatic autodigestion as well as subsequent inflammation.

In conclusion, the PAMORA-AP trial aims to investigate the potential beneficial effects of treatment with the PAMORA methylnaltrexone on disease severity and clinical outcomes in patients admitted with predicted moderate to severe AP. If successful, this trial will, for the first time, document the effects of a targeted pharmacotherapy in patients with AP.

## Trial status

Protocol Version 1.19, 2021.11.05. Recruitment has started in May 2021 in Aalborg, whereas the remaining sites have started recruitment in July 2021. So far, 16 patients have been included in the study. Last patient, last visit is expected by June 2023.

## Abbreviations

AE: Adverse event
AP: Acute pancreatitis
AR: Adverse reaction
eCRF: Electronic case report form
GCP: Good clinical practice
IAP/APA: International Association of Pancreatology/American Pancreatic Association
IMP: Investigational medicinal product
PAMORA: Peripherally acting μ-opioid receptor antagonist
PASS: Pancreatitis activity scoring system
PEG: Polyethylene glycol
SIRS: Systemic inflammatory response syndrome
